# Effect of chemotherapy and aromatase inhibitors in the adjuvant treatment of breast cancer on glucose and insulin metabolism—A systematic review

**DOI:** 10.1002/cam4.1911

**Published:** 2018-12-18

**Authors:** Kristian Buch, Victoria Gunmalm, Michael Andersson, Peter Schwarz, Charlotte Brøns

**Affiliations:** ^1^ Diabetes and Bone‐Metabolic Research Unit, Department of Endocrinology Rigshospitalet København Ø Denmark; ^2^ Department of Oncology Rigshospitalet København Ø Denmark; ^3^ Faculty of Health Sciences Copenhagen University København N Denmark

**Keywords:** breast cancer, chemotherapy, glucose, insulin, weight gain

## Abstract

**Introduction:**

Breast cancer (BC) is the most common cancer among women worldwide. With increasing survival rates, focus has expanded to long‐term adverse effects of adjuvant chemotherapy and/or aromatase inhibitors. Weight gain during chemotherapy has been well documented, but the underlying mechanisms remain unclear. A change in glucose and insulin metabolism is a possible consequence.

**Methods:**

We searched PubMed on the 4th of May 2018, and found eight articles that compared measurements of glucose and insulin before and after chemotherapy and/or aromatase inhibitors in woman with BC.

**Results:**

A general trend of increased glucose and insulin is seen and likely to be caused by weight gain and/or changes in body composition as a consequence of adjuvant treatment of BC.

**Discussion:**

Due to methodological limitations including short follow‐up times and small sample sizes, further studies are required to better describe metabolic consequences of adjuvant chemotherapy and/or aromatase inhibitors. Future studies could help identify patients in high‐risk of developing cardiometabolic disease after BC treatment.

## 1 INTRODUCTION

Breast cancer (BC) is the most common cancer among women worldwide, accounting for 25% of all new female cancer cases in 2012.^1^ Survival rates in Europe have increased through screening and improved treatment. However, despite the success, there might be unintended complications associated with adjuvant chemotherapy and/or aromatase inhibitors, since some women with BC experience adverse long‐term metabolic effects.^2^

Adjuvant or neoadjuvant chemotherapy, which is cytotoxic to tumor cells, is most often given as a combination of drugs. The most common regimens include sequential anthracycline‐cyclophosphamide and taxane (AC‐T); concurrent anthracycline‐cyclophosphamide and taxane (ACT); anthracycline‐cyclophosphamide without taxane (AC); cyclophosphamide, methotrexate, and fluorouracil (CMF); and docetaxel and cyclophosphamide (TC).^3^ Aromatase inhibitors are the first hormonal therapy medicine choice for postmenopausal women whereas tamoxifen, which is a selective estrogen receptor modulator (SERM), is the first choice for premenopausal women. The aromatase inhibitors stops the production of estrogen and acts by blocking the enzyme aromatase, which turns the hormone androgen into estrogen in the body, resulting in less estrogen and in turn impair growth of hormone‐receptor‐positive BC cells. The most prevalent types of aromatase inhibitors used in the clinic in treatment of postmenopausal women are anastrozole, exemestane and letrozole.^4^

It has been known since the 1970’s that women receiving adjuvant chemotherapy experience weight gain, commonly reported as 2‐5 kg but with great variance.^5^, ^6^, ^7^ Furthermore, a recent review emphasizes the important role of in particular body composition on overall health status of BC patients,^8^ as excess adiposity may increase mediators of not only BC recurrence but also of metabolic disease. Gadéa et al^9^ looked at publications on chemotherapy‐induced weight changes, concluding that no consensus existed on the cause of metabolic derangement pointing toward adipokines, insulin and insulin‐like growth factor (IGF) being possible factors. Aromatase inhibitors, used for treatment of hormone receptor‐positive BC, effect on lipid metabolism have been thoroughly described, demonstrating a negative effect on the lipid‐profile due to a lack of estrogen as opposed to tamoxifen which has an agonistic estrogen effect outside of the breast.^10^ Furthermore, a meta‐analysis from 2008 demonstrated an increase in cardiovascular events in patients treated with aromatase inhibitors compared to tamoxifen,^11^ and a recent study found that aromatase inhibitors increased the risk of diabetes mellitus.^12^ However, the specific effect of chemotherapy and/or aromatase inhibitors on glucose and insulin metabolism is poorly understood with only a limited amount of studies having focused on this.

Very few animal studies have examined the metabolic impact of chemotherapy; Story et al^13^ reported weight gain in dogs receiving carboplatin while another study found significant increases in total and LDL cholesterol in rats when treated with cisplatin and oxaliplatin.^14^ Pivotal studies on humans have linked alkylating agents to hypertension^15^ and increased cholesterol,^14^ but metabolic factors are rarely part of initial drug testing. Multi‐agent regimens and longer treatment duration may be factors resulting in weight gain,^5^, ^9^ but there is in general a lack of research on long‐term metabolic side effects.

Altogether, studies suggest that women receiving adjuvant chemotherapy and/or aromatase inhibitors for BC experience metabolic alterations giving rise to a phenotype associated with increased risk of developing cardiometabolic disease as well as BC recurrence.^9^, ^16^ The importance of improved understanding of long‐term impact as well as the underlying mechanisms is underlined by the fact that women who are free of disease after 5 years of tamoxifen treatment are more likely to die from non‐BC causes.^17^ However, no data exist on longer follow‐up times with regard to non‐BC causes of mortality.

The purpose of this systematic review is thus to assess the current available knowledge on impact of adjuvant chemotherapy and/or aromatase inhibitors in women with BC on metabolic alterations focusing on glucose and insulin metabolism.

## 2 METHODS

**Figure 1:**
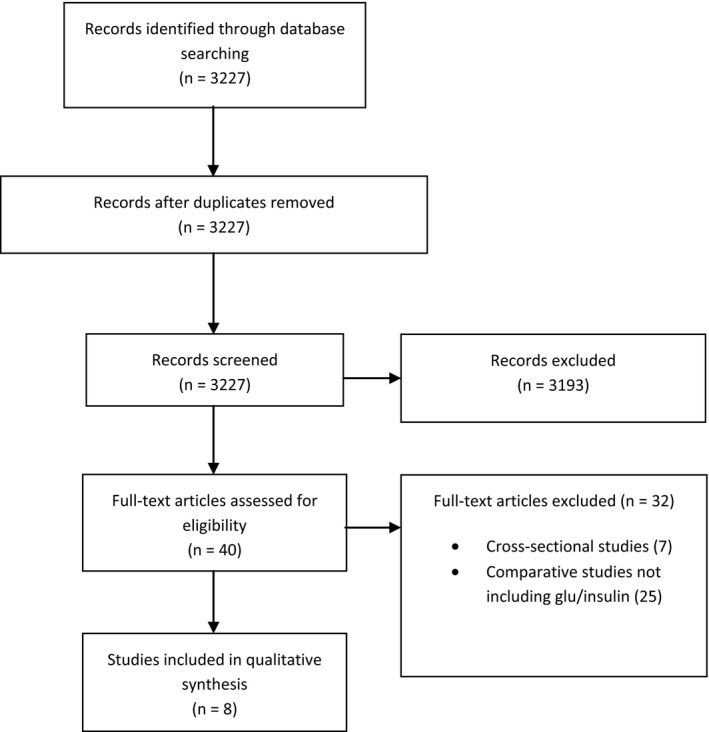
Flow chart depicting literature search

This review has been written according to the PRISMA guidelines. All articles describing the effect of adjuvant chemotherapy and/or aromatase inhibitors in women with BC and how it affected glucose, insulin and/or insulin resistance by comparing measurements before and after treatment were identified and included (Figure 1). No limits to follow‐up time or publication date were specified in the attempt to get as many relevant articles as possible. Exclusion criteria included non‐English articles, although relevant English abstracts for foreign language articles were assessed. Screening of articles was performed by reading titles, and if relevant abstracts, to find articles for full text evaluation.

We searched PubMed (Medline) on 4th of May 2018 with the following search terms:

(Breast Neoplasms[mh] OR Breast Neoplasm*[ti] OR Breast Neoplasm*[ot] OR Breast Carcinoma[ti] OR Breast Carcinoma[ot] OR Breast cancer[ti] OR Breast cancer[ot])
AND
(Oncological treatment[ti] OR Oncological treatment[ot] OR Antineoplastic Combined Chemotherapy Protocols[mh] OR Chemotherapy[ti] OR Chemotherapy[ot] OR Chemotherapy, adjuvant[mh] OR Adjuvant[ti] OR Adjuvant[ot] OR Chemoradiotherapy, adjuvant[mh] OR Chemoradiotherapy[mh] OR Chemoradiotherapy[ti] OR Chemoradiotherapy[ot] OR Neoadjuvant Therapy[mh] OR Neoadjuvant[ti] OR Neoadjuvant[ot] OR Antineoplastic Agents, Hormonal[mh] OR Aromatase Inhibitors[mh] OR Aromatase Inhibitors[ti] OR Aromatase Inhibitors[ot] OR Tamoxifen[mh] OR Tamoxifen[ti] OR Tamoxifen[ot] OR Raloxifen[mh] OR Raloxifen[ti] OR Raloxifen[ot])
AND
(Body composition[mh] OR Body composition[ti] OR Body composition[ot] OR Body Weights and Measures[mh] OR Diabetes[ti] OR Diabetes[ot] OR Dyslipidemias[mh] OR Dyslipidemia*[ti] OR Dyslipidemia*[ot] OR Fat percentage[ti] OR Fat percentage[ot] OR Glucose Intolerance[mh] OR Glucose Intolerance[ti] OR Glucose Intolerance[ot] OR Hypercholesterolemia*[ti] OR Hypercholesterolemia*[ot] OR Hyperglycemia*[ti] OR Hyperglycemia*[ot] OR Hypertriglyceridaemia*[ti] OR Hypertriglyceridaemia*[ot] OR Insulin[ti] OR Insulin[ot] OR Insulin Resistance[ti] OR Insulin Resistance[ot] OR Insulin Sensitivity[ti] OR Insulin Sensitivity[ot] OR Metabolic Disease[mh] OR Metabolic syndrome[ti] OR Metabolic syndrome[ot] OR Metabolism[mh] OR Metabolism[ti] OR Blood glucose[mh] OR Body Mass Index[mh] OR Metabolic syndrome[mh] OR Metabolic syndrome[ti] OR Metabolic syndrome[ot] OR Triglycerides[mh] OR Waist‐Hip Ratio[mh])

## 3 RESULTS

As shown in Figure 1, the search yielded in total 3227 articles, which were screened for eligibility, 3193 articles were excluded due to lack of information regarding metabolic factors during adjuvant chemotherapy and/or aromatase inhibitors of BC. Forty articles were further assessed for eligibility and 32 excluded due to either being cross‐sectional studies (N = 7) or not including measures of glucose/insulin (N = 25). The eight studies included in the systematic review are presented in Table 1.

**Table 1:** Details of studies examining glucose/insulin metabolism

	Oncological treatment	Follow‐up, mo	Breast cancer stage	Number of patients (pre‐/postmenopausal)	Mean age, y	Weight/BMI	Glucose	Insulin	HOMA‐IR
Prospective studies									
Dieli‐Conwright et al^18^	AC‐Taxol/TC/CT/AC/TCH	Mean 4	I‐III	86 (46/40)	48.2	↑	↑	↑	↑
Coskun et al^22^	N/A	6	II‐III	20 (5/15)	51	↔	↔	↑	↑
Alacacioglu et al^23^	TEC	4	II‐III	17 (8/9)	50.5	↑^c^, *	↔	↑	↑
Arpino et al^20^	AC‐Taxol^a^	6‐24 (median 14)	N/A	433 (175/258)	53.6	↑	↔	↔	N/A
Hickish et al^21^	FEC/FEC‐T	4.5‐6^b^	N/A	39 (N/A)	58.6	N/A	↑^d^	N/A	N/A
Chala et al^24^	FEC	6	IV	10 (0/10)	63.2	↔	↔	↓^e^	↔
Retrospective studies									
Guinan et al^19^	N/A	Mean 39.4	N/A	61 (31/30)	51.0	↔	↑^f^	↑	↑
Bicackli et al^25^	TAC	6.75	II‐III	104 (62/42)	47.4	↑	↔	N/A	N/A

TEC (docetaxel, epirubicin and cyclophosphamide), FEC (cyclophosphamide, epirubicin and 5‐flouracil), TAC (docetaxel, doxorubicine, cyclophosphamide) AC (doxorubicin and cyclophosphamide), fluorouracil, epirubicin and cyclophosphamide), AC‐Taxol (doxorubicin and cyclophosphamide followed by paclitaxel), TC (Docetaxel and cyclophosphamide), CT (carboplatin and paclitaxel), TCH (docetaxel, carboplatin and trastuzumab), FEC‐T (cyclophosphamide, epirubicin and 5‐flouracil followed by docetaxel).

a Most patients receiving chemotherapy only received this regimen, rest received undescribed combination including trastuzumab.

b After six cycles of chemotherapy, no time between cycles indicated.

c *P*‐value is shown as 0.05, but in the text the value is mentioned as not being significant.

d Only the subgroup receiving dexamethasone.

e OGTT at 120 min.

f HbA1c.

* *P* = 0.05 defined as NS by the authors.^23^

Adjuvant chemotherapy regimen varied between studies; four of the studies used a combination of an alkylating agent (cyclophosphamide or carboplatin) along with anthracyclines (doxorubicin or epirubicin) and taxanes (docetaxel or paclitaxel). Chala et al use a regimen of alkylating agent with anthracyclines and fluorouracil and Hickish et al vary between both of the abovementioned combinations. Two studies do not specify treatment. In three of the studies it is indicated that some patients received endocrine treatment, Dieli‐Conwright et al^18^ uses a regimen including trastuzumab in 6% of patients, Guinan et al^19^ state that patients may have received trastuzumab and Arpina et al^20^ followed chemotherapy with endocrine treatment in most patients without further clarification.

The eight articles were published between 2006 and 2016, with a prospective study design in six and a retrospective study design in the two remaining studies. The general trend of the studies was to use measurements from before the first cycle of chemotherapy and compare to measurements during or shortly after the last cycle. The follow‐up periods range from 3 to 40 months, but with only two studies having an average follow‐up time of more than 7 months. Four of the studies reported a significant increase in weight/BMI whereas three studies did not find a significant change in body weight (Table 1). However, likely due to the short follow‐up times, only marginal weight gain is reported, with the exception of Dieli‐Conwright et al^18^ who reported an increase of 5.5 kg.

Study size ranged from 10 to 433 patients, where only three studies included more than 50 patients. BC stage was not reported in three studies, where the remaining studies included stage I‐III patients, with only one study exclusively examining stage IV BC patients. The studies included both pre‐ and postmenopausal women, with the exception of one study exclusively including postmenopausal women and one study not specifying menopausal state. The mean age ranged from 48.2‐63.2 years.

Blood glucose or hemoglobin A1c (HbA1c) was measured in all studies. HbA1c is a measure of glycation of hemoglobin, and as red blood cells have a lifespan of up to 120 days, it represents a measure of the average blood glucose concentration during the past 8‐12 weeks. Most studies found no significant change from before to after adjuvant chemotherapy and/or aromatase inhibitors. However, two studies found a significant increase in glucose and HbA1c respectively^18^, ^19^ and one found a significant increase in a subgroup treated with dexamethasone.^21^

Insulin was measured in six studies and was significantly increased after adjuvant chemotherapy in four of these. Contrary to this Chala et al found a significant decrease in insulin at 120 minutes during an oral glucose tolerance (OGTT) test compared to before adjuvant therapy. Homeostatic model assessment of insulin resistance (HOMA‐IR) was calculated in five of the studies, and was significantly increased in four of the studies.^18^, ^19^, ^22^, ^23^

## 4 DISCUSSION

### 4.1 Summary and limitations

Although only a limited number of studies have looked at the impact of adjuvant chemotherapy and/or aromatase inhibitors on glucose and insulin metabolism in women with BC, it appears that a negative effect is the most common observation. The eight studies in focus do have severe limitations. The short follow‐up times make it difficult to predict long‐term consequences of metabolic changes. Likewise, are several of the studies small in sample size and some are retrospective studies.

The pre‐ and post‐treatment weight and/or BMI were reported in seven of the eight studies. Four of the studies reported a significant increase in weight and/or BMI in response to treatment.^18^, ^20^, ^23^, ^25^ The women included in the studies had a pretreatment BMI >25 kg/m^2^ which correspond with the fact that increased weight, is a risk factor of BC. However, none of the studies included a control group, thus no comparable background information including weight and BMI exist on aged‐matched women without BC.

Weight gain is common among aging women, especially during the menopausal transition. According to several studies, the average weight gain in women during midlife is approximately 0.4‐0.7 kg/y independent of initial body weight and ethnicity, however with a large individual variation.^26^, ^27^ This information is important to keep in mind when evaluating the effect of chemotherapy and hormonal treatment in women with BC.

The generalizability of the studies to current BC patients is limited by the differing treatment, with only four studies using regimens that reflect current practice with the combination of an alkylating agent combined with an anthracycline and a taxane. Furthermore, many studies lack detailed descriptions concerning treatment, especially with regard to endocrine treatment. The adverse effects on metabolism are poorly understood and rarely the focus in clinical or animal studies of chemotherapy effect in cancer. Finally, has weight gain not been clearly linked to any specific regimen or single chemotherapeutic agent.^9^

### 4.2 Duration of metabolic derangement

The long‐term effects of metabolic changes induced by chemotherapy are difficult to assess, but in a study of more than 3000 women Saquib et al^29^ found that only 10% returned to their pre‐BC diagnosis weight over a 6‐year period.

Among the studies on the glucose metabolism, only two have follow‐up periods of more than a year, with Guinan et al^19^ having follow‐up periods of up to 40 months showing an increase in HbA1c, insulin and HOMA. Arpino et al,^20^ on a much larger cohort found no significant change in glucose or insulin with an average follow‐up time of 14 months. Several studies have tried to examine if the negative effect could potentially lead to an increase in diabetes diagnoses. Lipscombe et al^30^ found an increase in diabetes incidence from 2 years post surgery. This is substantiated by other studies,^12^, ^31^ although some find no significant change in incidence.^32^

### 4.3 Potential mechanisms

The underlying cause of the metabolic alterations including changes in glucose and insulin metabolism is not clear and are most likely only part of the metabolic changes occurring as a consequence of adjuvant chemotherapy and/or aromatase inhibitors of BC patients. However, the previously mentioned weight gain and/or changes in body composition are likely to be factors contributing to the metabolic changes. Weight change during chemotherapy may be caused by treatment‐related side‐effects such as fatigue, physical inactivity, sarcopenia and altered appetite. The increase in circulating lipids^18^ could also be the cause for changes in glucose metabolism. Alternative mechanisms include reduced basic metabolic rate^33^ as well as changes in the circadian rhythm in response to chemotherapy.^34^

It is likely that certain subgroups may be at increased risk of unfavorable changes in body composition following chemotherapy. Previous studies have suggested that premenopausal women may gain more weight during treatment of BC than postmenopausal women^8^ and that women with low body weight at diagnosis may gain more weight at the start of treatment than overweight women.^35^ The weight gain is primarily of adipose tissue with a tendency to lose muscle mass also known as sarcopenic obesity.^8^

Adiposity may increase the risk of metabolic disease, and in recent years adipokines and primarily leptin has been investigated. Leptin is a hormone produced in the fat tissue and is positively correlated with body fat mass.^36^ Several studies have tested if adjuvant chemotherapy and/or aromatase inhibitors affects leptin production, and while most find no significant change^22^, ^23^ others find high leptin levels linked to tamoxifen as well as raloxifene use.^37^, ^38^ Akyol et al^39^ shows a significant increase in leptin during aromatase inhibitor and tamoxifen treatment, but also an increase in the antagonistic adipokine adiponectin making the clinical effect unclear. Low levels of adiponectin have been linked to increased BC mortality.^40^

Interleukins has been shown by Paz et al^41^ to be affected by chemotherapy, as they found that despite IL‐6 being increased and IL‐10 being decreased compared to controls before treatment; chemotherapy further enhanced this tendency. Another study found no change in interleukins, including IL‐6, during adjuvant chemotherapy and/or treatment with aromatase inhibitors.^24^ Fibroblast growth factor 21 (FGF‐21) was decreased during aromatase inhibitor and tamoxifen treatment suggesting a positive effect on metabolism.^39^ It is also worth noting that certain genetic polymorphisms have been linked to loss of fat free mass during aromatase inhibitor treatment,^42^ suggesting that a subgroup of patients could be more susceptible to metabolic adverse effects.

### 4.4 Consequences

With obesity being an independent risk factor for development of BC in postmenopausal women,^43^, ^44^ BC patients, as a group, are in a worse metabolic condition than a comparative group of women.^45^, ^46^ Therefore, a further metabolic derangement of this group of patients, with an otherwise high survival rate,^2^ could potentially cause cardiometabolic disease in the future. This is highlighted by cross‐sectional studies showing an increase in metabolic syndrome of postmenopausal BC survivors compared to healthy controls^47^ and increased visceral adipose tissue due to tamoxifen use.^48^ Cheney et al^49^ found that regardless of weight gain or loss, women receiving adjuvant chemotherapy and/or aromatase inhibitors for BC were likely to gain fat mass and lose lean body mass.

### 4.5 Prevention of adverse metabolic effects

Exercise training and dietary interventions have been suggested to alleviate or minimize treatment‐related side effects to BC. Recent systematic reviews and meta‐analysis have shown significant effects of physical exercise on anthropometric measures and quality of life.^50^, ^51^ The ENERGY study is the largest weight loss intervention trial among survivors of BC to date, including both dietary guidance and physical activity to promote weight loss over a 2 year period, resulting in a ~4% weight loss among elderly overweight or obese women.^52^ However, interpretation of most results from exercise and dietary intervention studies must be done with caution because of relatively poor quality of evidence, methodological limitations, low numbers and short follow‐up time etc Further well‐designed studies are therefore required to determine the impact of exercise and dietary interventions on treatment‐related side‐effects. Moreover, follow‐up studies are particularly required because of possible long‐term side effects of adjuvant treatment of BC.

## 5 CONCLUSION

Women undergoing medical treatment for BC, appear to become metabolically deranged to some extent. This is mainly evident by a small increase in weight and/or change in body composition, but the underlying mechanism has not yet been thoroughly described. Earlier explanations like overeating,^53^ seem unlikely on its own, due to the loss of lean body mass and development of sarcopenic obesity.^54^ A combination of fatigue, physical inactivity, altered appetite and sarcopenic obesity having a negative impact on especially the insulin and glucose metabolism appear to be the most likely explanation. The studies in this field are few and of varying quality, which is why larger studies over a longer duration is needed, as well as studies on patients using up‐to‐date BC treatment regimens. Because of observed treatment‐related changes in body composition, physical activity as also recommended during treatment and recovery, as it might indeed be beneficial for this patient group to overcome metabolic derangements.

## CONFLICT OF INTEREST

None declared.
